# The Carceral Shadow: Criminal Justice as a Determinant of Health and Challenges for Policymakers

**DOI:** 10.1111/1468-0009.70095

**Published:** 2026-05-24

**Authors:** RASHAWN RAY, KEON GILBERT

**Affiliations:** ^1^ University of Maryland; ^2^ The Brookings Institution and Saint Louis University

**Keywords:** criminal justice, social determinants of health, police violence, mass incarceration, racial health disparities, carceral health, Public Health Critical Race Praxis, health equity

## Abstract

**Policy Points**

- The criminal justice system functions as a primary social determinant of health in the United States, generating disproportionate physical, psychological, and chronic health burdens on Black communities and other marginalized groups.
- Policing structural barriers—including qualified immunity, police union contracts, and municipal financing of misconduct settlements—systematically shield law enforcement from accountability and divert public resources from health‐promoting investments.
- Evidence‐based interventions—including coresponder programs, community violence interruption, diversion initiatives, and Medicaid prerelease enrollment—demonstrate measurable improvements in public safety and health equity outcomes.
- Policymakers must adopt a health‐in‐all‐policies approach that reallocates resources from punitive criminal justice practices to upstream investments in housing, mental health care, youth employment, and community well‐being.
- Transforming criminal justice into a health‐promoting system requires cross‐sectoral leadership, rigorous research, and training reform that centers racial equity, officer well‐being, and community‐driven public safety models.

**Context:**

The United States incarcerates more people per capita than any peer nation, and its criminal justice system disproportionately impacts Black, Indigenous, and other communities of color. Police violence, mass incarceration, and carceral health crises each independently generate significant health burdens, while their intersection compounds longstanding racial health disparities. The COVID‐19 pandemic amplified these structural failures, exposing the lethal consequences of overcrowded facilities, inadequate health care access, and systemic racism embedded across criminal justice institutions.

**Methods:**

This perspective piece synthesizes peer‐reviewed literature, national surveillance data, and policy analyses to examine the criminal justice system as a social determinant of health. Drawing on the Public Health Critical Race Praxis framework, the authors identify mechanisms linking carceral practices to population health outcomes and evaluate both policy gains and persistent failures across policing, mass incarceration, and community health domains.

**Findings:**

Police violence, disproportionately directed at Black individuals—who are 3.5 times more likely than White individuals to be killed by police when unarmed—generates illness spillovers including elevated rates of chronic disease and mental health disorders in affected communities. Carceral settings function as breeding grounds for infectious disease, with COVID‐19 infection rates in some US detention facilities reaching up to 14 times higher than surrounding general populations. Policing structural barriers including qualified immunity, police union contracts, and municipal financing of misconduct settlements insulate officers from accountability and redirect billions in public funds away from health‐promoting services. Evidence‐based alternatives—including coresponder programs, diversion initiatives, community violence interruption, and Medicaid prerelease enrollment—demonstrate measurable reductions in crime, use of force, and health disparities.

**Conclusions:**

The criminal justice system is not a neutral arbiter of public safety but a potent and often detrimental determinant of health, particularly for Black and other marginalized communities. Dismantling the carceral shadow requires policymakers to adopt a health‐in‐all‐policies approach that reallocates resources upstream, holds law enforcement accountable, and centers rehabilitation over punishment. A fundamental reconception of public safety as public health is both a moral imperative and an evidence‐based strategy for advancing health equity and reducing the chronic disease, mental health, and mortality burdens generated by current carceral practices.

The health of a nation is profoundly shaped not only by its health care system but also by the myriad social, economic, and political forces that constitute its broader environment. In the United States, the criminal justice system, often viewed primarily through the lens of public safety and law enforcement, has emerged as a potent and often detrimental determinant of health, casting a long shadow over communities and individuals, particularly those marginalized by race and socioeconomic status. This perspective piece provides a critical opportunity to explore this nexus, demanding that state policymakers confront the complex interplay between carceral practices and population health outcomes. It will offer critical insights into past policy gains and persistent failures, propose actionable solutions, and consider the broader implications for social policy, training, research, and leadership in addressing these pressing challenges.

The criminal justice system's influence on health extends beyond direct physical harm, encompassing chronic stress, mental health crises, infectious disease transmission, and the erosion of community social capital.^1^ The COVID‐19 pandemic tragically magnified these existing disparities,^2^ exposing the lethal consequences of inadequate health care access, systemic racism, and overcrowded carceral facilities.^3^ Understanding this system as a public health intervention, or often, a public health failure, requires a critical examination of its historical roots, its contemporary manifestations, and its disproportionate impact on Black, Indigenous, and other communities of color.

The literature presented herein—highlighting police violence, racial disparities, and the systemic impacts on both communities and law enforcement personnel—underscores the critical role of prison culture and environment as determinants of public health and safety, emphasizing rehabilitative strategies within correctional facilities. This essay elevates an urgent need for states to adopt forward‐looking strategies that prioritize health equity and redefine public safety.

## Past Gains and Successes in Both Policy and Practice

While the narrative often focuses on the shortcomings of the criminal justice system concerning public health, it is important to acknowledge areas where progress, however incremental, has been made or where certain trends have improved, often due to broader societal shifts or targeted interventions. One notable, yet complex, success has been the significant decline in overall violent crime rates in the United States over several decades. The reported violent crime rate per 100,000 population steadily decreased from its peak in the early 1990s through 2020. Despite increases linked to COVID‐19 spillovers related to high unemployment and underemployment, violent crime has continued to fall and is substantially lower than in previous decades. In 2025, more than 30 cities saw their lowest murder rates in about a century.^4^ While various factors contribute to this trend—including economic improvements, demographic shifts, changes in policing strategies, and community‐led initiatives—a reduction in violent crime lessens exposure to trauma and injury, thereby contributing to public health. This decline has, in some contexts, allowed for a reevaluation of hyper‐punitive policies that characterized earlier eras.

Furthermore, there have been nascent movements toward criminal justice reform aimed at reducing incarceration rates and addressing systemic inequalities. Initiatives such as the “ban the box” movement (currently in more than 35 states), which seeks to remove questions about criminal history from initial job applications, aim to improve employment prospects for formerly incarcerated individuals, thereby addressing a crucial social determinant of health. At the same time, research documents that young Black and Latino men may consequently face a perceived criminality penalty because employers assume they are more likely to have a criminal record.^5^ Similarly, some states have explored alternatives to incarceration for nonviolent offenses, diverting individuals to drug treatment programs or mental health services, recognizing that these issues are often public health concerns rather than purely criminal ones. Diversion programs reduce recidivism by roughly 50%.^6^ These diversions, while not uniformly implemented or universally successful, represent a conceptual shift toward addressing root causes rather than solely relying on punitive measures. Recently, there has also been meaningful progress in reducing the overall incarceration rate. After decades of sustained increases, the US state and federal prison population has declined notably since its peak. Bureau of Justice Statistics data indicate that the incarcerated population fell by roughly 25% between 2009 and 2022, with particularly notable reductions for drug‐related offenses.^7^ Researchers project that this downward trend may steepen further in coming years as reform‐oriented prosecutorial practices, revised sentencing guidelines, and bipartisan decarceration legislation take hold.^8^ Neal and Kilmer^9^ further document the shift in criminal justice responses to drug offenses away from incarceration and toward diversion, treatment, and harm reduction. These developments represent genuine policy successes, though the United States continues to incarcerate at rates far exceeding those of peer nations, and racial disparities in incarceration remain severe and largely unchanged.

The growing recognition of the impact of mental health and substance use disorders within the criminal justice system has also led to positive policy developments. Crisis intervention training for law enforcement officers and mental health co‐responder programs with mental health experts, aimed at de‐escalating encounters with individuals experiencing mental health crises, have been adopted in many jurisdictions. For example, in examining the state of New Jersey's ARRIVE Together co‐responder program, Ray found that mental health professionals responding to calls for service with law enforcement decreases use of force and increases utilization of mental health services.^10^ While the effectiveness and breadth of implementation vary, these programs signal a greater understanding that certain interactions require a public health, rather than solely law enforcement, response. Similarly, some jurisdictions have implemented drug courts and mental health courts, which seek to connect individuals with treatment services rather than traditional incarceration, offering a pathway to recovery and improved health outcomes. Recent rigorous research further substantiates these programs’ promise. Dee and Pyne found that a community‐based crisis response approach in Eugene, Oregon, significantly reduced crime and emergency service demand.^11^ Studies of mobile crisis response teams document that pairing mental health clinicians with law enforcement produces measurable reductions in arrests and improved health service utilization.^12^ Notably, in Durham, North Carolina, the implementation of a co‐responder model produced a striking and counterintuitive outcome: a marked increase in 911 calls following program launch. This increase reflected growing community confidence that calling 911 during a loved one's mental health crisis would result in trained, non‐coercive intervention rather than arrest—a crucial indicator of restored public trust in crisis response systems.^13^

However, it is crucial to qualify these successes. The decline in violent crime, for instance, has not necessarily translated into a reduction in the most egregious forms of state‐sanctioned violence or a mitigation of racial disparities. Police killings have increased significantly over the past two decades, and the heightened national attention following the murder of George Floyd in May 2020 did not produce a durable reduction in lethal force. According to Mapping Police Violence, more than 1,100 people were killed by police in both 2022 and 2023—among the highest annual counts in the modern tracking era—demonstrating that police reform efforts have thus far failed to translate into population‐level declines in police lethality.^14^ Furthermore, bipartisan police reform at the federal level seems nonexistent, indicating that even agreed‐upon reforms face significant political hurdles.^15^ Political partisanship seems especially salient with the increase in immigration enforcement across the country. In 2025, more than 540,000 people were arrested as part of US Immigration and Customs Enforcement (ICE) raids.^16^ These limited gains underscore the profound challenges that remain, particularly concerning issues of equity and the systemic nature of criminal justice as a health determinant.

## Failures and Continuing Problems

Despite some limited progress, the criminal justice system in the United States continues to manifest significant failures that profoundly undermine public health, particularly for marginalized communities. These failures are deeply rooted in historical injustices and perpetuated by contemporary policies and practices. In the following discussion, we focus on six social problems that continue to establish criminal justice as a determinant of health.

### Disproportionate Police Violence and Its Health Consequences

The starkest failure lies in the persistent and disproportionate use of force, including lethal force, by law enforcement, particularly against Black individuals.^17^ Although Black people constitute roughly 13% of the US population, they make up 31% of all people killed by police and about 40% of those killed while unarmed and not attacking. Moreover, Black people are 3.5 times more likely to be killed by police when they are not attacking and do not have a weapon. This racial disproportionality is not an anomaly: Across various subgroups, including youth and young adults and lower and higher incomes, Blacks, compared to Whites, are significantly more likely to be killed by police. Altogether, Black men have “a 1 in 1,000 chance of being killed by police over the life course”.^18^ As noted earlier, violent crime has been plummeting over the past few decades, indicating that police violence is often not a direct response to community crime rates but rather a systemic issue. This disconnect undermines arguments that increased police killings are a necessary evil to control crime.

At the same time, many people juxtapose police killings alongside the disproportionate rates of violent crime victimization that Black communities endure. In 2023, Black Americans constituted an estimated 53.8% of all homicide victims despite representing roughly 13% of the population.^19^ Comparable disparities exist across other serious violent crime categories. These dual realities—disproportionate victimization by both interpersonal violence and state force—are not in tension but rather compound each other as evidence of systemic failure. In fact, there is little correlation between cities that experience high rates of violent crime and those that experience high rates of police killings (Mapping Police Violence).^14^ Still, Black communities experiencing high rates of violent crime have the most urgent need for effective, accountable, and trust‐building law enforcement. It is precisely because both interpersonal violence and police violence fall most heavily on Black communities that the reforms proposed in this essay—accountability structures, de‐escalation training, and community co‐production of public safety—are most critical. Social service investments and policing strategies and practices will have their greatest beneficial and harmful impacts in communities that simultaneously face the twin challenges of prevalent public safety threats and prevalent police misconduct.

Beyond direct mortality, police violence and exposure to pervasive policing practices have severe “illness spillovers.”^20^ People living in areas with frequent police killings are more likely to suffer from obesity and high blood pressure. Men's mental health suffers in neighborhoods with high rates of stop‐and‐frisk and force. Women in these same neighborhoods report higher rates of diabetes, blood pressure, and obesity. This evidence establishes a significant association between the criminal justice system's actions and chronic health conditions, transforming policing into a significant public health hazard.

Ray's research^21^ sheds further light on the insidious ways systemic racism impacts health, even in seemingly benign public spaces. His study on why Black men might hesitate to exercise in “White spaces” revealed that people often overestimate the number of Black people they see in public space. This social psychology of criminalization amplifies other assumptions people might make about a Black man running down the street of a White neighborhood, often leading to the Black man being viewed as a threat due to deeply ingrained stereotypes. This perception, fueled by racial bias in policing and society, contributes to Black men's hesitancy to engage in physical activity in certain areas, thereby contributing to “countless health risks” such as higher rates of obesity, heart disease, and stroke among Black individuals.^22^ This highlights how the pervasive shadow of criminal justice and racial prejudice extends even to daily health choices.

### Sociohistorical Roots and Systemic Impunity

Disproportionate police violence against Black and Indigenous bodies extends even before the founding of the United States on American soil. Enslavement, socially sanctioned lynching, and the prison industrial complex all contribute to mortality and morbidity gaps between racial groups. The Public Health Critical Race Praxis (PHCRP) framework emphasizes how these origins continue to criminalize Black males, limit healthy identity formation, and lead to higher rates of killings due to biases, discrimination, lack of accountability, and abuses of power.^17^, ^22^ The killings of George Floyd, Breonna Taylor, Daunte Wright, Elijah McClain, and Tyre Nicholes are examples of a system “imbued with institutional and cultural failures that expose civilians and police officers to harm.”^15^

Regarding mass incarceration, the Violent Crime Control and Law Enforcement Act of 1994 increased the number of law enforcement officers and exacerbated the prison population, coinciding with “stop‐and‐frisk policies and a rise in stand‐your‐ground laws that disproportionately disadvantaged Black Americans and led to overpolicing.”.^15^ Biases in policing and the criminal justice system more broadly are not always conscious or on the micro‐level. Rather, biases can be engrained in organizational culture on the meso‐level and manifest upstream from systematic policies and laws that disadvantage some groups while advantaging others. For example, despite similar rates of drug use and distribution between Black and White individuals, there are significant disparities in who is arrested, convicted, and incarcerated for drug‐related crimes, underscoring how systemic bias dictates outcomes. Troublingly, some neighborhoods have such poor health and social outcomes that some Black men in state prisons have lower mortality rates than Black men not incarcerated.^23^ The organizational culture of policing is further shaped by documented racial attitude disparities within law enforcement itself. A 2020 Pew Research Center analysis of a nationally representative survey found that White and Black police officers hold starkly divergent views on fundamental questions of racial equality: 92% of White officers—but only 29% of their Black colleagues—believed the United States had already made the changes needed to ensure equal rights for Black Americans.^24^ These divergent worldviews have measurable behavioral consequences. Donahue^25^ documents how the politics of policing—including officers’ partisan identity and racial attitudes—shape patrol behavior and use‐of‐force decisions in the field. Recent analysis by Ba and colleagues^26^ similarly finds that political diversity within agencies predicts variation in use‐of‐force patterns. The institutional embeddedness of these attitudes is further evidenced by research documenting that White police officers played a prominent role in the organizational infrastructure of partisan political campaigns.^27^ Taken together, this evidence underscores that bias in policing is not solely an individual problem but an organizational one, reinforcing the importance of systemic reforms to training, screening, and departmental culture.

### Ineffectiveness and Misallocation of Resources

In local communities, the traditional model of policing often proves inefficient in addressing core public safety needs. Nine out of every 10 calls for service are for nonviolent incidents. Yet, social service staff are often left on the sidelines while police respond to calls that may be better suited for the skill sets of other experts. Furthermore, homicide clearance rates are alarmingly low (less than 40%), as are rates for other serious crimes, indicating that current policing models are not effectively solving crimes or ensuring justice for victims.^28^ In addition, inefficiency is exacerbated by racial disparities in service delivery. People living in predominately Black and Latino neighborhoods wait significantly longer for law enforcement and other first responders to arrive. This indicates a systemic failure to provide equitable public safety services, directly impacting community well‐being and trust.

### The Health Burden on Law Enforcement

The failures of the system are not limited to the communities it polices. There is a significant mental health crisis among police officers themselves.^29^, ^30^ A high percentage (roughly 80%) experience depression, anxiety, and chronic stress; recurring memories; family problems; anger; and sleep issues. Alarmingly, 16% report thoughts of suicide and 15% report substance use disorders. Despite these struggles, nearly 90% do not reach out for help or seek therapy due to the stigma associated with mental health issues within law enforcement. This indicates a profound systemic failure to support the well‐being of officers, which in turn can contribute to burnout, poor decision‐making, and negative interactions with the public.

### Systemic Obstacles to Accountability: Qualified Immunity and Police Union Contracts

A critical failure of the criminal justice system in protecting public health is its entrenched resistance to accountability, largely shielded by legal doctrines and union contracts.^31^ A primary obstacle is qualified immunity, which inhibits people's ability to sue police and other government officials for violated civil rights and due process. Consequently, law enforcement is often insulated from civil liability for misconduct. Recently, Vice President JD Vance called for “absolute immunity” for border patrol and ICE agents. In turn, more than 500,000 people were arrested and detained by ICE, one‐third of whom did not have a criminal record and a sizable percentage of US citizens or legal residents. There are reports of profiling based on skin tone, phenotype, and accent. Since January 2025, more than 30 people have died while in ICE custody.^16^

This lack of accountability extends to the financial burden of misconduct. From 2015‐2019, the 20 largest US municipalities spent more than $2 billion in civilian payouts for police misconduct.^31^ Crucially, these funds largely come from general city funds, not police department budgets, meaning the funds could be spent on social and public health services to improve health outcomes. Compounding this, police union contracts, particularly from the Fraternal Order of Police, are deeply embedded in law enforcement. They frequently shield officers from accountability for misconduct, making it difficult to discipline or terminate “bad apples.” This organizational structure creates a cultural environment where problematic officers can remain in uniform, undermining public trust and perpetuating harmful practices that directly impact community health.

### Health Crises in Carceral Settings: The COVID‐19 Pandemic and Beyond

The COVID‐19 pandemic served as a stark and tragic magnifying glass, exposing and exacerbating the existing health crises within the carceral system, disproportionately impacting already vulnerable populations. Black people, who are overrepresented in the criminal justice system, have been profoundly affected by the virus, facing higher rates of contraction, inadequate health care, and increased mortality. This inequitable impact extends well beyond hospitals, penetrating deeply into correctional facilities where structural conditions amplify vulnerability. Visher and Eason note that “prison culture and environment are essential to public health and safety.”^3^ However, traditional prison life is often fraught with difficulty and can be deeply traumatic. Incarcerated individuals face deprivation of liberty, separation from family, loss of self‐worth, and high levels of stress, leading to mental health disorders and difficulty in rejoining society. The environment in most US prisons is simply “not conducive to positive change,” making it exceedingly difficult for individuals to successfully reintegrate into communities.^3^

Carceral settings, by their very nature, are breeding grounds for infectious diseases. Overcrowding, poor sanitation, and limited access to quality health care create an environment where viruses can spread rapidly and lethally. The pandemic revealed horrifying infection rates within prisons and jails across the country. At various points during the height of the COVID‐19 pandemic, the follows statistics compounding on the prison population^32^:

- A state prison in Ohio reported approximately 2,000 COVID‐19 cases, accounting for nearly 20% of the state's total diagnoses at one point.
- In Arkansas, a maximum‐security prison housed about 40% of the state's COVID‐19 diagnoses.
- The infection rate in the Washington, DC, jail population soared to 14 times higher than that of the general population in the city.
- Federal prisons saw a COVID‐19 diagnosis rate that, while seemingly minuscule at 0.45%, was nearly double the percentage observed in the general US population. If the national diagnosis rate mirrored that of federal prisons, over 660,000 more Americans would have been diagnosed and an additional 30,000 would have died.

These alarming statistics underscore the profound failure to protect the health of incarcerated individuals and the ripple effect this has on broader public health. COVID‐19 deaths in prisons reveal a system ill‐equipped to provide even basic, let alone specialized, medical care, resulting in unnecessary suffering and death for both incarcerated people and correctional staff.

In summary, the criminal justice system, far from being a neutral arbiter of safety, acts as a primary determinant of health in the US, perpetuating racial inequalities, inflicting direct physical and mental harm, consuming vast resources inefficiently, and even harming its own practitioners.

## Potential Solutions: Policies, Strategies, Practices

Addressing the entrenched failures of the criminal justice system as a determinant of health requires a multifaceted approach involving significant policy shifts, strategic reallocations of resources, and changes in practice. Policymakers are uniquely positioned to spearhead these transformations.

### Reimagining Public Safety and Reallocating Resources: The “Defund the Police” Conversation

The concept of “defund the police” is often misunderstood, but, at its core, represents a critical strategy for improving public health by reallocating resources. Police department budgets must be “fiscally responsible and shift funding to focusing on solving violent crime, while simultaneously reducing use of force on low‐income and racial/ethnic minority communities.”^15^ Police departments often consume a substantial portion of municipal budgets. Research documents that police spending as a percentage of general funds can be around 40%. A portion of these funds can be redirected from law enforcement to community‐based health, housing, education, and violence prevention programs. States can invest in upstream solutions that address the root causes of crime and poor health.^33^ It is equally important that advocates and researchers advancing these resource reallocation arguments attend carefully to political framing. The “defund the police” slogan, however well‐intentioned, proved politically toxic to some audiences—including older residents of communities of color who simultaneously support robust community investment and effective, humane policing in their neighborhoods. In many respects, many residents in predominately Black communities are seeking a qualitatively different relationship with law enforcement—one that resembles the humanity, care, and concern they often witness occurring in predominately White communities. Researchers and advocates are most effective as coalition partners when they attend to the full range of community priorities and communicate in terms that build rather than fracture the coalitions necessary for durable reform. This includes funding the following:

- *Mental health and crisis response teams*: Nonpolice units trained to respond to mental health crises, substance use disorders, and homelessness, reducing potentially violent encounters and connecting individuals with appropriate care.^10^
- *Community violence interruption programs*: Evidence‐based programs that treat violence as a public health issue, using credible messengers to mediate conflicts and prevent retaliation.^34^
- *Youth employment and school‐based interventions*: A robust experimental literature documents that upstream youth investments simultaneously reduce violence and address the mental health consequences of violence exposure. Heller demonstrated in a randomized trial that summer jobs programs significantly reduced violence among disadvantaged youth in Chicago.^35^ Complementary evidence from school‐based, trauma‐informed group interventions shows lasting reductions in violent behavior and improvements in mental health among young people exposed to community violence.^36^, ^37^ A longer‐term follow‐up by Abdul‐Razzak and colleagues^38^ confirms sustained behavioral gains, underscoring that school‐based behavioral science programs represent a high‐return investment for violence prevention and youth mental health.
- *Affordable housing and social services*: Investments in stable housing, accessible health care, and social support networks that reduce stress, improve health outcomes, and decrease involvement with the criminal justice system.^39^, ^40^ Phoenix's Office of Homeless Solutions and Little Rock's affordable housing program are models at the local level.

### Implementing Equitable Policing Policies and Practices

Existing research suggests a series of concrete measures to reduce police use of force. We list them below for brevity.

- *Mandate data collection and transparency*: States must require comprehensive and disaggregated data collection on all instances of use of force and death involving law enforcement, including demographic information of those involved, circumstances, and outcomes of investigations. This transparency is crucial for identifying patterns and holding officers accountable.
- *Repeal “stop and frisk” laws and end zero tolerance policies*: These practices disproportionately target marginalized communities and are major contributors to community distrust and chronic stress. Eliminating them aligns with the PHCRP principles of decriminalization.
- *Reform qualified immunity*: Many policing scholars advocate for the removal of qualified immunity to hold officers directly responsible for misconduct and require them to obtain professional liability insurance the way other occupations such as doctors, plumbers, lawyers, and electricians do. This shifts the burden of financial liability from taxpayers to the officers and their departments, creating a strong incentive for early identification and accountability for problematic officers. In 2021, the state of Colorado passed the Enhance Law Enforcement Integrity Act, which requires police officers found to have illegally violated someone's civil rights to pay 5% or up to $25,000 of the civil settlement for misconduct. Second, advocates recommend shifting civilian payouts away from general tax money to police department insurance policies. This would ensure that police budgets directly bear the financial consequences of misconduct, forcing departments to prioritize prevention and accountability. This also prevents municipalities from solely making individual officers responsible for misconduct rather than the department that frequently has an organizational culture that of abuse.^31^
- *Create national standards for training and de‐escalation*: There are currently no national standards for training law enforcement in the United States. Consistent, high‐quality de‐escalation training (currently less than 10 hours compared to 50 hours of firearm training) could be implemented to reduce use of force.^15^
- *Require body‐worn cameras (BWCs) with strict usage policies*: BWCs can increase accountability and provide objective evidence in cases of misconduct.^41^ Storage issues can inhibit departments from leaving BWCs on continuously. Strict usage policies can ensure that officers are using them correctly.
- *Implement civilian review boards with disciplinary power*: Establishing independent civilian oversight bodies that have the authority to investigate complaints, review disciplinary actions, and recommend policy changes can significantly enhance police accountability and community trust.
- *“Bad apple list” and “good apple protections”*: A “bad apple list” would track officers with patterns of misconduct, preventing them from simply moving to another department. Conversely, “good apple protections” would safeguard officers who report misconduct or adhere to ethical practices, fostering a culture of accountability from within. Texas, California, and New York have all passed versions of a “wandering officer law” preventing officers from transferring to or being hired by another department if they were fired or under investigation for misconduct.^42^

### Prioritizing Officer Well‐Being and Professional Development

Addressing the mental health crisis among police officers is not only an ethical imperative but also a public safety strategy. Key solutions include mandatory mental health counseling for officers to destigmatize mental health treatment, mandatory housing subsidies for officers to live where they work (closer to home and closer to community members), mandatory bias training to address social dominance and far‐right extremist views as well as better psychological assessment for these outcomes during the evaluation process, and enhanced training in de‐escalation, crisis intervention, and cultural competency. The Lab for Applied Social Science Research at the University of Maryland helped develop a virtual reality training program for Jigsaw, a subsidiary of Google.^43^ This platform integrates artificial intelligence data from law enforcement to move beyond traditional methods, allowing officers to troubleshoot situations in the field, especially ones with heightened ambiguity, where biases are most likely to surface. Advances in this program can incorporate and measure physiological reactions of officers (e.g., heart rate, cortisol levels) to different challenges, leveraging this data to make informed decisions about which officers patrol the streets and which sit behind a desk at the station, ensuring officers are well‐suited and supported for their roles.

### Targeted Interventions for Carceral Health Crises and Rehabilitation

Considering the vulnerabilities exposed by COVID‐19 and the documented challenges of prison life, states must implement specific policies to safeguard health within correctional facilities and address the underlying systemic issues, moving beyond incapacitation to rehabilitation.^3^ Given the health challenges of prisons, states should prioritize the immediate release of incarcerated people who pose minimal public safety risk. This includes individuals in jails for pretrial detention, those held on nonviolent charges, people approaching parole eligibility, and those over 60 years of age. As seen with actions by Maryland Governor Larry Hogan to expedite releases, this strategy is crucial for reducing population density and preventing disease spread. Both lawmakers and organizations like the Rainbow PUSH Coalition and the Justice Policy Institute have advocated for such measures. There should also be rehabilitative programs and increased health care access for incarcerated people. Diversion programs clearly work to put people on a pathway to upward mobility. This can occur farther downstream in prison. By implementing these comprehensive policies, states can begin to dismantle the carceral shadow and cultivate a system that genuinely promotes public health and safety for all.

A critical and underutilized lever for improving health outcomes among people with criminal–legal system involvement is Medicaid. Expanding Medicaid to prerelease enrollment and ensuring seamless transition services upon release can connect returning citizens with sustained behavioral health care, substance use disorder treatment, and primary care at a moment of acute vulnerability. Chin and colleagues document the population health implications of Medicaid prerelease and transition services for incarcerated populations, finding meaningful improvements in continuity of care and health outcomes.^44^ Complementary analysis by Statchen and colleagues in the *Journal of Health Politics, Policy and Law* details the opportunities and limitations of Section 1115 substance use disorder waivers as a vehicle for expanding Medicaid coverage to justice‐involved populations.^45^ As states expand Medicaid and implement innovative Section 1115 waivers, ensuring that justice‐involved individuals are enrolled and retained in coverage represents a high‐priority policy lever for advancing health equity and reducing recidivism.

## Implications for Broader Social Policy, Training, Research, and Leadership in the Field

The transformation of the criminal justice system from a health determinant to a health promoter necessitates profound shifts that extend far beyond law enforcement agencies themselves. These implications touch every aspect of social policy, reshape professional training, demand new avenues of research, and require bold, visionary leadership. The following sections outline some of these important implications.

### Implications for Broader Social Policy

Recognizing criminal justice as a determinant of health compels policymakers to adopt a “health in all policies” approach, which includes the following.:

- *Investing in upstream solutions*: Redirecting resources from reactive, punitive measures to proactive investments in social determinants of health. This includes expanding access to quality education, affordable housing, living wage jobs, nutritional food, and comprehensive health care, including mental health and substance use disorder treatment. These investments directly reduce the conditions that lead to criminal activity and involvement with the justice system, fostering healthier communities.^33^
- *Reimagining public safety*: Shifting the paradigm from relying primarily on police to address all societal problems to a more holistic, community‐led public safety model. This involves empowering communities to design and implement their own safety solutions, often rooted in restorative justice practices and violence prevention. It means continuing to divert individuals dealing with mental health crises from the criminal justice system, promoting better community outcomes.^3^
- *Mitigating the impact of incarceration*: For individuals who are incarcerated, policies must focus on reducing the health harms of imprisonment. This includes improving health care access within correctional facilities, ensuring humane conditions, and implementing robust reentry programs that connect individuals with health services, employment, and housing upon release to prevent recidivism and promote long‐term health.^3^ Furthermore, policy must address the systemic barriers facing formally incarcerated people, such as the exclusion from federal aid programs, which hinder their ability to reintegrate and contribute economically.

### Implications for Training

The skills required for effective, health‐promoting public safety extend far beyond traditional law enforcement training.

- *Law enforcement training reform*: Moving away from military‐style training that emphasizes force and control toward curricula focused on de‐escalation, crisis intervention, cultural humility, trauma‐informed care, and community engagement. Officers need to be trained as community guardians, problem solvers, and first responders to social and health crises, rather than primarily as enforcers. This includes specialized training on mental health, substance use disorders, and disability awareness. Advanced trainings that leverage technology, virtual reality, and artificial intelligence are key.^42^ These trainings are mobile, cost‐efficient, leverage a small geographic footprint, and allow officers to train based on normative social interactions rather than worst‐case scenarios.
- *Correctional staff training in rehabilitative practices*: As part of the shift toward a rehabilitative focus in prisons, correctional staff must be trained in evidence‐based therapeutic methods, particularly cognitive behavioral therapy, to effectively facilitate cognitive behavioral communities.^3^
- *Expansion of nonpolice first responder training*: Developing and adequately funding training programs for mental health professionals, social workers, and community mediators to serve as primary responders for nonviolent calls for service^10^ creates a diversified public safety workforce better equipped to address the complex needs of communities.

### Implications for Research

A commitment to evidence‐based policymaking requires a robust research agenda.

- *Quantifying health impacts*: More rigorous, longitudinal research is needed to comprehensively quantify the direct and indirect health impacts of various criminal justice policies and practices—from policing strategies to incarceration rates—on physical, mental, and social well‐being across different demographic groups. This must specifically include the health burden of infectious disease outbreaks within carceral settings and their spillover into surrounding communities.
- *Evaluating alternative models*: Research must rigorously evaluate the effectiveness of nonpolice response models, community‐led violence prevention programs, and alternative justice interventions (e.g., restorative justice) in improving health outcomes and reducing crime. This includes cost‐benefit analyses to inform resource allocation.
- *Evaluating prison‐based rehabilitative programs*: Research must focus on assessing programs that actually rehabilitate rather than perpetuate incarceration. This type of research is crucial to identify effective strategies for promoting positive change within prisons and successful reintegration into society.
- *Conducting police officer well‐being studies*: Further research into the occupational stress, mental health challenges, and effective interventions for law enforcement officers is critical, informing policies that support their well‐being and foster a healthier policing culture. What type of training and what type of hiring are more likely to lead to healthier outcomes for the public and police officers?

### Implications for Leadership in the Field

Transforming the criminal justice system into a health‐promoting entity demands courageous and collaborative leadership at all levels of state government and within communities. We lay out four critical themes that will lead to better health.

- *Visionary state leadership*: Members of Congress, governors, state legislators, and agency heads must advance a clear vision that frames public safety as public health, prioritizing prevention and equity over punishment. This requires political will to challenge entrenched interests and invest in innovative solutions.
- *Collaborative governance*: Leaders must foster cross‐sectoral collaboration, bringing together justice officials, public health experts, community organizers, advocacy groups, and affected individuals to co‐create policies and practices, particularly related to implementing innovative organizational approaches that address problems like safety, staff burnout, and prisoner grievances.
- *Accountability and transparency*: Leaders must commit to transparent data reporting, robust oversight mechanisms, and holding individuals and institutions accountable for misconduct and systemic failures. This builds trust and ensures that reforms are genuinely implemented.
- *Community engagement and empowerment*: Authentic leadership involves listening to and empowering communities most affected by criminal justice practices. This means supporting community‐led initiatives, respecting lived experience, and ensuring that policymaking processes are inclusive and representative. This implication is for academics. We need scholars to lead with their research and the policy implications of their scholarship. It is important to be a narrative setter and policy informer by engaging with the media, writing op‐eds in national publications, and testifying before state and federal governments on topics ranging from public health and racial equity to criminal justice reform. Academic research must directly inform and influence public policy and legislation, bridging the gap between scholarship and decision‐making to advance health equity.

## Conclusion

The evidence is clear—the criminal justice system in the United States functions as a significant and often detrimental determinant of health, particularly for Black communities and other marginalized groups. The disproportionate rates of police violence, the collateral health consequences extending from chronic disease to mental health crises, and the systemic inefficiencies and biases ingrained in the system, exacerbated by structural barriers to accountability like qualified immunity and union contracts, present an urgent public health crisis. Policymakers have a critical opportunity and responsibility to address these challenges. By reallocating resources from traditional policing to community‐based health and social services and implementing equitable policing policies, the carceral shadow of criminal justice can be illuminated and addressed. Addressing the health crises within prisons and jails through decarceration, improved health care, and robust rehabilitative programs, combined with broader investments in health and economic infrastructure for marginalized communities, is paramount. These solutions are not isolated; they require broad shifts in social policy, comprehensive training reforms, dedicated research to inform best practices, and courageous leadership committed to justice and health equity. The path forward requires a fundamental shift in how we conceive of safety and community well‐being, moving away from punitive responses to societal problems toward a holistic, public health approach. By embracing these challenges, policymakers can transform the criminal justice system from a source of disease burden and premature mortality into a genuine partner in building healthier, more equitable communities for all.
